# A Work Environment Under Pressure: Psychosocial Job Demands and Resources Among Saturation Divers

**DOI:** 10.3389/fpubh.2022.765197

**Published:** 2022-04-28

**Authors:** Siri Romsbotn, Ingrid Eftedal, Jonas Rennemo Vaag

**Affiliations:** ^1^Department of Psychology, Faculty of Social Sciences and Educational Sciences, Norwegian University of Science and Technology, Trondheim, Norway; ^2^Department of Circulation and Medical Imaging, Faculty of Medicine and Health Sciences, Norwegian University of Science and Technology, Trondheim, Norway; ^3^Faculty of Nursing and Health Sciences, Nord University, Bodø, Norway; ^4^Faculty of Social Sciences, Nord University, Bodø, Norway

**Keywords:** saturation diving, baropsychology, psychosocial work environment, mental health, isolated and confined environment

## Abstract

Saturation divers work and live under high physiological and social demands for weeks on end. Even though physiological research has contributed insights to the work conditions of saturation divers, research on the qualities of the divers' psychosocial work environment is lacking. This study aimed to explore which job demands and resources are viewed as characteristic among saturation divers working within an isolated and confined environment. Based on data from 6 in-depth semi-structured interviews, template analysis was applied to map unique characteristics. By using the theoretical framework of the job demands-resources model, we found that the work environment in saturation diving was characterized by shifting demands and big contrasts, requiring adaptability in each individual diver. One major demand described by the informants was *an unpredictable future*, somewhat due to the changes in the oil and gas industry. Another important demand was the *conflict between family and work/leisure* when committing to work for extended periods in isolated environments. The *monotony* that characterizes the work environment is a challenge that must be managed. *High wages, periods of leisure*, and *a prestigious job* provide external motivation, while personal resources such as *mental endurance and flexibility, a willingness to learn*, and *keeping up small personal routines*, may benefit the divers' mental health. This is also affected by the *quality of team climate*—with features such as being *sociable and considerate*, having *a dark sense of humor* and having *trust* in one another.

## Introduction

Divers have played an important role in the development of the offshore oil and gas industry in the North Sea since its beginning. The diving activity was necessary for the implementation of the industry and has contributed to societies economic income. Accumulated research has led to an improvement in saturation diving procedures. Today, saturation diving can be performed safely and efficiently, but there are still many questions to be answered (1). A knowledge status on offshore diving (2) indicates that most research on saturation diving to this day are physiological studies investigating how the human body reacts to, or adapts to living and working in an extreme hyperbaric environment [e.g., (1–6)]. Yet, there is a shortage of studies investigating possible psychological effects of this type of diving (2). Saturation diving was introduced in the North Sea in the 1970s [(7), p. 47–50]. Because the divers don't return directly to the surface, saturation diving requires a comprehensive support system, including pressurized living chambers, diving bells, and life support systems usually installed on a Diving Support Vessel (DSV). The pressurization, or the so-called “blowdown”, takes place in the saturation chambers on the DSV, where the pressure is increased to match the pressure they experience at working depth. This system is where the divers live when they are not working on the construction site on the seabed (1), and is therefore equipped accordingly. On the vessel, many people serve to support the life and work of the divers in the pressurized chamber system, including dive managers, dive supervisors, life support technicians, and life support supervisors. These people control what the divers do in the water and the chamber at all times, what they breathe, what they eat, supply them with personal necessities, etc., to keep them alive and comfortable on very few square meters (7, 8). As explained in Kiboub [(9), p. 2] the divers are usually organized in four teams of three men, each team on a shift of 12 h overlapping the other teams, whereas, they live together in three or six-man chambers, depending on the DSV. The teams rotate to ensure continuous “in-water” work activity.

At or near to the seabed the divers perform tasks such as assembling, maintaining and disassembling offshore rigs, wells, and pipelines (10), and tasks that require more precision and maneuverability than a remotely operated vehicle (ROV) can manage, e.g., hyperbaric welding tasks (1). Though saturation diving is a very procedural and carefully planned job, divers must also get around challenges, such as lose bottom material that can reduce the visibility to near zero in extreme cases, as well as ocean currents giving the divers additional physical strain (11). The maximum duration the diver stays saturated in the North Sea is 21 days in the Norwegian sector, and 28 days in the UK sector down to depths of 180 meters of sea-water (msw) (12, 13), including the time needed for decompression back to surface pressure.

## Working in Isolated and Confined Environments

Abraini et al. (14) characterized the work environment of saturation divers as physically and socially extreme. Physically, because the divers are exposed to an increased pressure related to the depths of the dives. Heliox is the preferred gas in saturation diving, since hyperbaric air has higher breathing resistance and can induce nitrogen narcosis. In deeper dives, below 180 msw, the divers are at risk of developing high-pressure nervous syndrome (HPNS) a neurological syndrome characterized by headache, tremor, vertigo, nausea, and moderate changes in cognitive functioning (1, 2, 14). Socially, because saturation diving requires long-term confinement in saturation chambers for weeks on end, the environment in hyperbaric saturation can be boring due to the lack of stimulation, absence of privacy, and social interaction with a restricted number of people, as well as a reduction in the satisfaction of basic human needs such as feelings of personal significance, and need for affection (14).

The saturation divers' work environment can be described as an isolated and confined environment (ICE), wherein the divers experience prolonged isolation, and confinement. ICEs exist in different forms, e.g., deployment on nuclear submarines, long-distance sailing voyages, underwater laboratories, polar expeditions, and long-term missions in outer space (15). Psychological studies on humans living and working in such environments for extended periods of time have provided a greater understanding of how environmental, individual, and social factors influence human behavior. In such environments, the living and working conditions of the ICEs provide a natural laboratory for studying human behavior. Studies like these are important in order to promote successful adaptation and wellbeing, as well as minimizing potential negative consequences of living, and working in these “unnatural” environments (15).

ICE-related research on submarine missions have listed stressors such as lack of privacy, interpersonal tension, scheduling, assignment of duties and leadership style, as well as homesickness and feeling isolated (16). A review by the NASA Human Research Program has listed, amongst others, experience, emotional stability, motivation, job satisfaction and adaptability as personal factors needed in order to cope with stressors in ICE environments. On the team level—group identity and common cultural and personality traits are indicated as preferable (17).

## Psychosocial Work Environment and the Job Demands-Resources Model

The quality of the psychosocial work environment has been shown to be important for the individual employees' wellbeing, productivity, and health (18, 19). One model that balances the negative and positive job characteristics and comprises a broad definition of psychosocial work environment is the Job Demands-Resources model (JD-R model). The JD-R model is a heuristic and flexible theoretical framework that considers how job characteristics influence individual wellbeing and work performance. This is done by integrating motivational and stress research. The model implies that physical, psychological, and contextual factors of the psychosocial work environment can be categorized into two broad categories, job demands and job resources (20). The resources and demands may be located in social and interpersonal relations, the tasks that are performed, in the individual employee, or in the organization at large. The combination of these two categories leads to two fairly independent psychological processes that influence employees' performance and wellbeing: a health impairment process and a motivational process (20). The health impairment process assumes that in poorly designed jobs, the job demands function as initiators of a process of health impairment fostering burnout or physical or psychological strain. Whereas, job resources initiate a motivational process, hence fostering growth, learning, and development (21).

In the JD-R model, the job demands are defined as psychological, physiological, social, or organizational aspects of the work situation that requires skills or effort. Examples of job demands are high risk perception, heavy lifting, and interpersonal conflict. Meanwhile, the motivational process assumes that the job resources are described as the factors that contribute to reducing physiological and psychological costs of the job demands; by helping the employee achieving work goals, and stimulates to personal development, learning, and growth (22–24), and may generate work engagement and organizational commitment (23). Examples of job resources may be the perceived job control, social support from colleagues or management, and performance feedback. The model assumes that high job demands and limited job resources can result in burnout as this form of working conditions lead to an undermining of employees' motivation and depletion of energy (25). The relationship between the job demands and resources and work engagement has been shown not to vary significantly. Hence, the JD-R model can be used irrespective of the type of occupation (23), for instance saturation diving.

The work environment in saturation diving consists not only of the job characteristics related to the procedural tasks performed on the seabed, but it also includes periods of living in a confined and isolated saturation chamber system with their colleagues. When pushing the boundaries of human experience, as it's done with the living and working conditions of saturation diving, it is reasonable to believe that there are challenges involved that are different from those encountered in “common” occupations. Psychological studies on commercial saturation diving are scarce, and the aim of this study was to use the framework of “The Job Demands-Resources Model” to map the demands and resources that is prevalent among saturation divers. This leads up to the research question: “*Which unique demands and resources are viewed as characteristic among saturation divers working within an isolated and confined environment?”*

## Methods

The present study follows a qualitative and exploratory design suited to obtain an in-depth account of individual's own unique experiences and perspectives (26), as well as rich descriptions of unique events, and giving voice to views that are rarely heard (27).

### Informants

The informants were recruited by snowball sampling, where one of the participants functioned as a ≪gatekeeper≫(28) for access to the other informants, with a primary focus of obtaining insight into the working conditions for today's commercial saturation divers. Numbers provided through communication with the industry indicate that there are around 250 commercial divers in the North Sea during a year. Our sample consisted of six male certified commercial saturation divers with an average of 14 years (range 9–17 years) of experience of saturation diving. The sample consisted only of men due to a vast predominance of males in the saturation diving industry. The informants were all actively diving or had been saturation diving as their main source of income the last years. Due to the transparency within the very small community of saturation divers, further personal information about the informants is not disclosed.

### Data Collection

Before the data collection, the study received approval from the Norwegian Centre for Research Data. In all, six in-depth interviews were completed following a semi-structured interview guide (29). The interviews lasted ~80–90 min and were conducted over a period of 2 months (November 2020 to December 2020), all recorded using a digital voice recorder. Due to the COVID-19 pandemic and the fact that the informants were in different countries the interviews were carried out digitally using Zoom Video Communications. The interviews were divided into different topics. To get a thorough contextual understanding of the working day as a saturation diver the first part of the interview revolved around the diver's experiences and background and their general everyday working conditions (e.g., their motivation for becoming a saturation diver, years of experience, type of tasks performed, etc.). Further, the questions aimed to get an understanding of the specific job demands the divers face in their daily lives, and what kind of personal and job-related resources are helpful to master challenges and demands of their working life (e.g., issues relating personal characteristics to master this line of work, different type of challenges, social cohesion, etc.). Furthermore, the interviews were recorded and transcribed. Transcriptions were done thoroughly and as “true” to the audio file as possible (30) to avoid overlooking valuable information or statements. Transcriptions were done word-for-word (verbatim), including non-verbal utterances (e.g., pauses and irony, etc.), and indications of striking words.

### Analysis

The data analysis was carried out using Excel and Word (31) to structure and analyze the data systematically, as it comfortably handles large amounts of data. This was done in line with the steps of Template Analysis (32): familiarization; preliminary coding of data, clustering emerging themes, defining an initial coding template, applying the initial template to further data and modification if necessary, and finalizing the template into the full data set. The method was chosen because it allows for definition of some themes in advance of the analysis, together with a flexible coding structure that allows for an in-depth exploration of the richest aspects of the data (33). Hierarchical coding is a key feature of the template analysis, and the coding process is often done according to predefined themes. For this study, the *a priori* coding was done based on the two broad themes of *demands* and *resources*, using the JD-R model (20). The data was coded line-by-line (34), bearing in mind a potential adjustment or expansion of the initial template if needed, while remaining open to the data. After applying this initial template to the first three interviews it seemed befitting to proceed and finalize the analysis based on these two themes. Further, passages and paragraphs were reread and sub-themes and concepts related to the higher-order themes were revealed. When comparing the codes from the different interviews some themes and concepts were mentioned frequently, whilst others were disclosed more indirectly. Due to the different nationalities of the informants, some of the citations chosen to validate the findings of the study were translated from the spoken language into English by the researcher.

## Results

Compared to “regular” 9-5 jobs, saturation divers start a job assignment when stepping onto the dive support vessel (DSV) and end when they return to shore. Except for the time the divers spend working at the construction site on the seabed, the rest of the time is spent with their team either in transport and preparation and/or eating, sleeping, and living on very few square meters in the chamber for 21–28 days. A working life as a saturation diver, therefore, encompasses a lot of different factors. One of the informants highlights the breadth of the factors affecting how the divers experience their job:

*It's almost like a psychological experiment having three to six men living together in 8-12 square meters for four weeks. I think all the psychosocial factors are… they should be shed light on. Not only the diving but related to the personalities… It's an equation of many factors… The personalities of the divers, the different work environments, the diver's point of view, diver welfare, the different contractual relationships you work under, if you are a full-time employee or freelance, if you can provide for your family or maintain your daily life. They all affect the way you experience your working day or your perception of the job*.

In the following, the categories described to be unique to the psychosocial work environment of a saturation diver today are presented. As the categories in the following section are highly related to one another, they should be viewed as dynamic rather than exclusive entities. The categories will be presented in line with Table 1, keeping in mind the findings are related to the main template; *demands* and *resources*.

**Table 1:** Potential stressors and demands/protective factors and resources.

**Categories**	**Sub-categories**	**Citations**
1. An unpredictable future	a. Job Insecurity b. Lack of foresight	“We call it the Greta Thunberg experience. [laughs]”
2. Family/work conflict	a. Work/family conflict b. Family as a resource	“It's no secret that many relationships dissolve in this job. It's common among divers. And I get it. And I think the work situation plays a major role in it…”
3. Monotony		It's like Groundhog Day. Every day in there is the same.”
4. External resources	a. High wages and leisure b. A prestigious job	“The time off and the money, no doubt. The best part of this job is not working. Yeah.”
5. Personal resources	a. Mental endurance/flexibility b. Interested in learning c. Forming personal routines	“I usually tell people who want to start sat diving to leave their brain on the outside, what I mean is you can't think about your family or worry about getting injured”.
6. Team climate	a. Being considerate/sociable b. Interpersonal trust c. Dark Humor/rough social tone	“The thing you have to understand is that when you are in that chamber, you are not in control of your life, you are not. You are relying on people to look after you.”

### An Unpredictable Future

Although all the informants in this study had succeeded in making saturation diving their primary occupation, it became clear that the future in saturation diving is somewhat unpredictable. They explain that this line of work is challenged with a lack of predictability concerning future assignments, as well as the uncertainty of the future of saturation diving. This unpredictable future was considered to be one of the main challenges affecting the working lives of the divers.

#### Job Insecurity

The global supply and demand of fossil fuels highly affect the working situation of the saturation divers. During the interviews, it became clear that low oil prices and the current trend toward less use of fossil fuels leave the divers feeling insecure about their future in the industry. Most saturation divers in the North Sea are day raters, there are few positions, and many divers are eager to do this job. Three informants emphasize the high salary as a factor that contributes to their perceived job insecurity.

*Yeah, so basically, it's the current trend toward a… to not use fossil fuels. That puts tremendous pressure on our industry. And it's made people feel very insecure about their line of work. Because… and then obviously you get something like the Covid comes along where work is slowing down as it is, and this has made it.. this year for me financially has been terrible. So really it's probably to do with common… people's perceptions of what we do. I mean, you must have read about Norway's oil shame, so you know ultimately the oil has given this country so much, and a lot of us are very proud too. So…we often meet people who are very against what we do. And that is always a concern. The trend, I mean I'm flying with SAS today, and they are talking about electric planes, you know so.. you look into the future and you see this is.. you are always going to need divers. It's a different work. But saturation diving is very much orientated towards oil and gas, and the reason why it's been successful and well paid is because oil is worth a lot of money. But if people stop using it, and oil is not worth any money you know you are no longer… I wouldn't take a pay cut to do the dangerous job I do, the reason is that it's decent pay. (…) So it's a concern for all of us, is the fact that you know people are used to living on good money. We call it the Greta Thunberg experience*.^*^laughs^*^

The work environment of saturation diving was also described as very competitive, especially when the crude oil prices are low and there are few assignments. Since most saturation divers are not on contracts and therefore not secured a position on the DSV there is a lot of pressure to perform, especially for the young divers.

#### Lack of Predictability

The saturation diving industry is project-based. Compared to other offshore businesses there is no regular crew change in saturation diving. The different national regulations, the depths of the sea, and the different clients would make a regular crew change very expensive and less efficient. Therefore, when the divers leave the DSV after an assignment and return home, they often don't know when they are expected to work again, or whether they are coming back at all. All of the divers interviewed in this study pointed to the lack of predictability in assignments to be one of the main challenges of their work situation, and that assignments often are sprung on them.

*For me the biggest challenge… It must be the insecurity…, not being able to plan… The unpredictability, not insecurity. The unpredictability of the work situation. Not being able to plan ahead. (…) We've never been able to plan a holiday*.

### Family-Work Conflicts

Another major challenge most of the informants experienced was balancing this line of work with family life. As mentioned above, foresight in assignments seemed to be a challenging factor also affecting the work-home balance negatively in terms of not being able to plan when you have time off and preparing the family before new assignments.

*So I guess… they tend to spring the work on you as well, they tell you we are going to work in a week, so at the end of the week you start getting ready and they delay it a week. Aah… and you are like Jesus Christ… you know what I mean because when they are putting your life on hold and you told your kids… I have to tell my daughter. I say to her sweetheart, I'm going back to work next week and I‘m trying to mentally prepare her. And she goes like “dad, are you going back to work?”, I say “oh no I am home for a week” and then the next call it's back on again*.

Other challenges regarding the work-home balance mentioned in the interviews were difficulties adjusting to life at home after an assignment, being away for weeks at a time, and missing out on life events such as birthdays and funerals. The informants also mentioned that stress to the family/work balance has forced many divers to quit their jobs or ending marriage/relationships because doing both did not harmonize.

#### Family as a Resource

Although the strain of balancing family and work-life was mentioned as a major challenge, three informants also mentioned their family or life partner as a resource in balancing their work-life as a saturation diver in terms of giving the life back home some structure, as well as receiving support.

*Uhm no… I think when I first started it.. how can I say. Meeting my wife was a really important step to managing my work-life experience. I have a Norwegian wife, and I think without her my life would be very different. She has been quite a rock, or a hand break even. I don't know if you spoke much about it, certainly when I started… take a 20-year-old male and lock him away for 28 days in a chamber with other men. All they are going to talk about are women, cars, and how much money they are going to make. And then take him out of that chamber with a substantial amount of money and send him home. He has got no wife, he has got no kids, it's the middle of the week and you can imagine how destructive that can be. You know you have been locked away, you are not yourself. I'm telling you. You don't do any… as a diver when you get out of that chamber, it takes you a week until you are thinking right again. You know you can be really snappy, you can be very aggressive. Some people, you know, I certainly see a lot of obsessive-compulsive behavior amongst divers and certainly… I have experienced it so as I've got older it's gotten easier. Certainly having a stable home-life is a massive help. But I would say that the thing is with diving you get a lot of free time and you've got a lot of money, and that is not always a good thing. I feel personally I manage it well now, you know my home life is incredibly busy with* ^*^number^*^ *children, and yeah my wife and I spend a lot of time together. I don't… you know… I feel like when I am away, that when I am at home I want to be with my family so… that's good but I have colleagues that are totally opposite. They come back and they spend most of their leave drinking and yeah not living a healthy lifestyle, so it's a real mixed kind of… (…) I certainly know guys that don't manage it well. Like I said, a big part of it is a stable home-life*.

### Monotony

Another major challenge to this occupation is the monotony of living in the chamber system. One of the informants compares the working life to Groundhog Day (a movie where a weatherman finds himself living the same day over and over again). The informant explains;

*It's like Groundhog Day, every day in there is the same. There are very few highs and lows. (…) The faster the time goes mentally, the better*.

Being able to handle the monotony is considered necessary in order to last in this profession. He continues;

*Every day in here is a Monday. Monday morning every day. And when I am back home every day is a Saturday, sometimes a Friday. That's how I see it. You need to handle the monotony. If you can't handle that, you won't last very long, at all*.

To fill the time in the chamber, the informants spend time reading, watching movies on the iPad, or sleeping. As one of the divers explains “*There is an offshore saying; more sleep, less trip.”* Three of the informants explained that they have been studying both bachelors' and masters' degrees during their off-time in the chamber in order to make the time pass, and to have a plan B or a way out of the industry.

### External Resources

Some external resources, namely high wages, long periods of leisure, and the prestigious appraisal of the job, appeared to be important for the decision to become a commercial saturation diver and to sustain this line of work.

#### High Wages and Leisure

Historically, commercial saturation diving is known for its high wages. During the interviews, it became apparent that the high wages were considered an extremely important factor in the divers working life. All the informants in this study mentioned high wages as one of the main reasons for becoming a saturation diver, along with the long periods of leisure.

*Hehe, the money and the leisure. That's about it. That's the reason I started diving in the first place. Working offshore where the money is good, and you have a lot of time off. As easy as that. That's my motivation*.

After several years in the saturation diving industry, wages and leisure appeared to be the same two factors considered to be the best part of this occupation. Most of the informants also mentioned excitement as a factor when deciding to become a saturation diver. Yet, compared to leisure and pay, the excitement is not as present as it was in the beginning, or not even present at all. Two of the informants also mentioned money as a factor making it difficult to change course in terms of career path.

#### A Prestigious Job

Further, another factor described in the interviews was the inaccessibility of saturation diving. It became clear that it is challenging getting a job in saturation diving, so getting a job offshore was prestigious.

*Becoming a sat diver was quite prestigious at the time. Especially in low circles, if that makes any sense to you? It was more… 50/50, the economic side was a driving force, if it was a badly paid job no one would do it, but it was the kind of yeah…, it was a hard job getting a well-paid job as a saturation diver. There were divers and there were DIVERS. If you made it in the North Sea that was quite a big deal, so yeah*.

### Personal Resources and Coping Mechanisms

Among personal qualities important to succeed in this profession, or to endure the high job demands they are constantly exposed to, three personal resources or coping mechanisms were considered useful; mental *endurance/flexibility*, the *interest in* learning and *forming personal routines*.

#### Mental Endurance/Flexibility

The informants described their work environment as not only physically but also mentally demanding. Five informants compare saturation diving with the military. Three informants reported that many divers obtain their certificate as saturation divers, don't come back after their first assignment. They point out that this job is not suited for everybody. When describing the personal resources important in saturation diving, five informants mentioned mental endurance or flexibility, or even being mentally detached as helpful in managing the strain of the rough work environment.

*You've got to be robust, you know you can't be… it's not the place for soft people or you know… You need to be tough, it's a little bit like the military in that way, you need to be physically and mentally durable, I'm not saying you have to be super tough or super fit. But I am saying… you have to be durable, you have to be able to tolerate… you have to have endurance – mental endurance. You have to be able to put up with shit that you wouldn't put up with in normal life, and you have to not be.. yeah… I'd say that the best characteristic is being durable*.

Further, two informants highlight mental detachment or the ability to shift the focus away from potential dangers in the job and being away from their families for a prolonged period as useful in managing this job.

*I usually tell people who want to start sat diving to leave their brain on the outside, what I mean is you can't think about your family or worry about getting injured*.

#### “Keen”/Interested in Learning

Being interested in how things work, willingness to learn, curiosity, or being “keen” are described as a personal characteristic often seen in the divers, as well as a characteristic the divers prefer in their colleagues.

*Being curious… you know, interested in learning. Always trying to figure out how things work. I see it in many of my colleagues, as well as in myself. I remember when I was a little boy and I got something for Christmas this year I got this pinball machine… a small one. There was no time to sit down and play, the first thing I did was take the screwdriver and start to pick it apart bit by bit to see how it really looked like. And when I have talked about this to my colleagues, many of them are like “yeah I did that too”. Being curious in doing everything, because you have to sort of… There is always something you have to learn.. always something new…You have to want to know how things work and.. yeah the curiosity, I think, has to be the most important characteristic*.

The informants also explain that even with the saturation diving courses you are not fully educated. To become a good diver and earn the other divers' trust you need to understand what you are doing, and if you are unsure, you always ask a more experienced diver. One informant also mentions that those who are not interested in learning everything usually don't make it in this industry.

*You have to ask questions! Don't think about trying to sort things out on your own. Never be afraid to ask! Everyone… There are so many things to this job that you have to learn. If a new diver doesn't ask any questions… I'll start to worry*.

#### Forming Personal Routines

Two of the informants pointed out that many saturation divers tend to make up their own routines in the chamber as a coping mechanism. These routines become very important for a good work situation; at times maybe too important if they don't manage to stick to it. This remark was confirmed by two of the other informants describing their own small, but very important routines in the chamber, or bringing their own equipment to work. Whether or not the informants were able to perform these routines were explained to potentially determine if the day was going to be good or not. The same routines were considered insignificant in their daily lives at home.

*I think… you know one of the key things is everybody has these little sorts of routines that can be quite important when you go in sat, and things like personal space so… in our system you've got a small box in the living area, that's your space you know, and I think that can be quite important… that you sort of manage that well. And that everybody respects that, but yeah no it's all these small things, really. I like… I try to make myself less reliant on those things. Sometimes you can become a little too focused on it and if one of those things isn't there, it's like the end of the world you know. If like… if I run out of sort of cardamom tea or whatever, that can be a big one so I got to make sure I've got enough of that. So yeah that is really it's being able to do the small things I think that makes you feel that you're not missing out on huge chunks of your life*.

### Team Climate

As the divers live and work closely for weeks at a time it's not surprising that the team composition of the assignments was argued to be a very important factor in their work situation. The composition of the dive team was described as having the potential to be either a demand or a resource. Small things that would not annoy you in the beginning, can become quite frustrating after weeks in isolation.

*When you have only been there for a week, things are still easy. The fourth week on the other hand. You have this guy biting on the spoon or eating with his mouth open. You want to kill him, you know. Ultimately, it's the small things you do, like in a relationship, that either make it or break it*.

The informants explain that the teams are carefully assembled by the diving supervisor and offshore construction manager (OCM) to be as even and efficient as possible. Three informants point out the difficulty of assembling teams that everyone is pleased with.

#### Being Considerate/Sociable

Most informants mention the comradery and working with divers they get along with as one of the most important factors for a good work situation. The ability to be companionable, sociable, and considerate was interpersonal characteristics they considered important.

*Hmm… Uhm… It's important to be sociable. To be socially competent. You have to know how to communicate with the other guys… That you understand the others [the divers], and fit in with the team. The job you do in the water, that's the part you can learn. You have to be willing and interested in the job, but that is definitely not the hardest part. When you've learned that, it is the part you know… the daily life of being in the chamber, and the decompression when you sit still maybe for a week or so, all day long. Uhm.. So that's maybe… Being a nice guy, that the other divers like you. I think that is the most important, presuming you have those abilities… you are interested in this field of work. Yeah*..

#### Interpersonal Trust

Moreover, trust was frequently mentioned as an important factor by the informants throughout the interviews regarding social cohesion, especially in the water. In order to conduct the job efficiently, the divers need to trust one another, and the supervisor, as well as the system around.

*It's the combination of the things… The job itself… Anyone could learn how to perform the different tasks, but when you are working in the water you need to trust everyone around you that they know what they are doing. You are very reliant on… and vulnerable to the mistakes of others. So you have to trust the system. Trust.. and sort out your insecurities. And focus on the job*.

The importance of the trust issue becomes evident when the informants describe situations working with new, or less experienced saturation divers. In situations like this, the divers explain that they must make sure that everything is done properly to ensure their own safety as well as the safety of their fellow divers. Lack of trust between team members was described as a challenge to the perceived safety in their job performance.

*Like I said, you've got to trust who you are in the water with. That is very important. Trust is built up over years of experience and sometimes… you know it can be quite tough because you want to make sure that things are done yourself if that makes sense, but.. then you need to have that trust element, uhm… to have complete cohesion as a team, really*.

#### Dark Humor/Rough Social Tone

The work environment in saturation diving is described as male-dominated and rough by most of the informants. Some informants highlighted the humor and social tone in this industry to be characteristic of this line of work. One informant linked this “gallows humor” to coping mechanisms, using humor for levity.

*It's still quite a bit of a male-orientated environment, there aren't many women that work offshore, there aren't any female sat divers that work with us. So it's a very, very male… environment. Other guys I've worked with are ex-military. There is a real macabre black.. dark humor we call it. You know the things you would joke about at work you would never ever talk about at home with your wife or society, so you can imagine living with six… five other men for twenty-eight days, the conversations can sometimes become quite coarse. Hehe. That's what men are like. So you know it's… its… I found it a challenge going into a normal work environment where I had really you know… there is a difference between having a laugh and then there is being careful not to say something that will offend someone. Offshore diving, it's a dangerous job and people use humor as levity, so you tend to joke about stuff. I mean I have got friends of mine that have been badly, badly injured to the point where they can never ever work again, and you… I've been in the chamber with them riding out for four days. They had to get to a hospital because they were crushed, and if you are super serious all the time you gotta laugh, cause if you don't then you know If you don't find… even if it's dark humor, uhm… I don't know… you can't take it too seriously you got to have a little bit of… yeah you got to be able to take the rough with the smooth sort of thing*.

## Discussion

**Figure 1:**
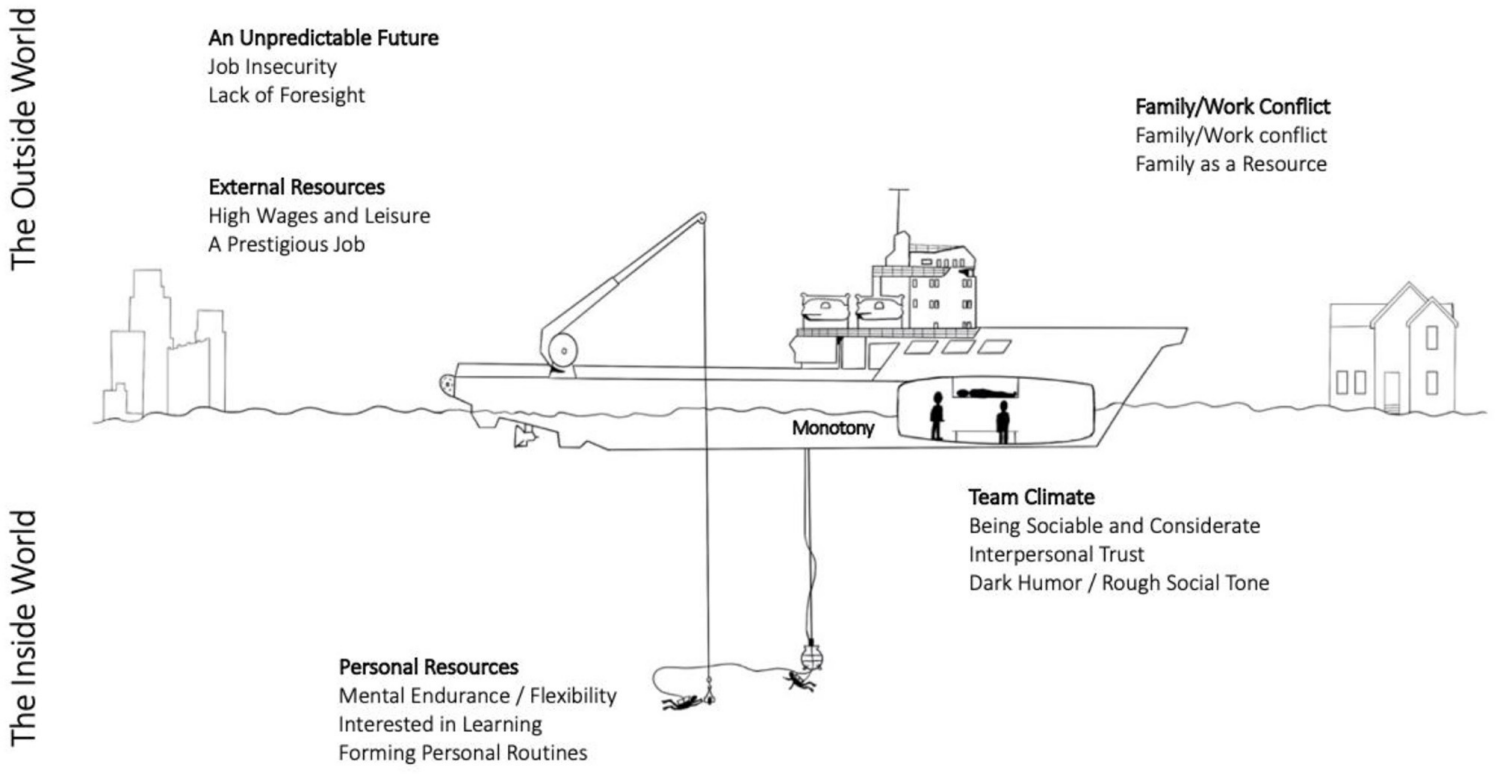
Overview of the personal, interpersonal and external demands and resources in saturation. The figure shows the different personal, interpersonal and external job demands and resources, and illustrates where they are located.

The purpose of this study was to gain an understanding of the psychosocial work environment of today's commercial saturation divers. This was done by mapping the unique job demands and resources that are characteristic of isolated and confined work environment in saturation diving. Among the many job demands such as physical strain, unfavorable working hours, risks and hazards, etc., the biggest challenges pointed out by the informants were *job insecurity*, and the *lack of predictability* related to changes in the oil and gas industry. *Work-family conflict* was another challenge as the divers spend 21–28 days away from their families and friends. The *social strain* due to living in an isolated and confined environment, along with *monotony* were mentioned as challenges of being confined to the saturation chamber system. Personal resources such as *mental endurance, being interested in learning*, a *sense of dark humor*, as well as *creating personal routines* were considered helpful in managing the daily life during saturation diving assignments. The interpersonal resources that were considered to be important were the reflected in the quality of the *social climate*, which depended on the *team harmony and trust* in one another. Additionally, the informants highlighted the external and organizational factors of *leisure and high salaries*, and a *prestigious job* to be the most important factors for job engagement and persistence, (see Figure 1) for an overview.

### Personal and Interpersonal Resources and Demands

According to the informants, According to the informants, personal resources determine how the divers adjust to the challenges they experience. This way, personal resources serve as coping mechanisms in managing the high demands and the big contrasts in the working life in saturation diving. The results indicate an active process of enduring the different demands by changing their focus away from stressor, using humor as levity, and crafting their off-time to experience control and meaning.

Although danger was not explicitly expressed to be one of the main demands, some of the personal resources still seem to function as a buffer toward this aspect of the job. The results of this study indicate that mental detachment or the ability to selectively shift the focus away from the inherent dangers of the occupation, or challenges due to prolonged periods away from their families are helpful to the divers. Similarly, research on detached mindfulness—that is the ability to observe internal events without responding to them—found that trusting luck and practicing detached objectivity would reduce proneness to worry (35). A Swedish study investigating daily uplifts and effective coping strategies for the hassles in the working lives of military veterans suggested that similar coping strategies—e.g., living in a bubble where risks aren't acknowledged and distancing oneself from emotions—may limit stress reactions under constant exposure to various stressors (36). This means of limiting the perception of danger is also seen among another group experiencing ICE; Israeli submariners, as a strategy of “optimal denial” (37).

Along with the shift of focus, the use of dark humor and gallows humor was commonly used as relief in saturation diving. Gallows humor is frequently seen among individuals who face traumatic, stressful, or life-threatening situations regularly, e.g., medical personnel, police, and firefighters (38), and is described as a method for maintaining sanity in situations that are experienced as “insane” (39). The use of humor is a well-known coping mechanism that acts as a buffer against negative stress effects (40), and has been linked to coping strategies such as distancing oneself from the stressor (41). Our results of this study are in accordance with previous studies indicating that humor reinforces resilience against distress (40–43). This effect was also seen in a study investigating coping mechanisms among the Israeli submariners that found humor accompanied with cynicism to be one of the most common coping mechanisms (37).

It has been suggested that organizations characterized by great demands are more likely to foster resilience resources to manage a potentially damaging process (44). Space flight experts have defined resilience during a space mission to involve mental endurance or the ability to sustain when being exposed to stressors such as monotonous tasks or poor lighting, along with recovering from acute stressors, such as unscheduled space walks (45). Similarly, to the findings of this study, mental endurance was one of the main personal resources highlighted by the divers for coping with demands such as the monotony of the working days in saturation, and the procedural tasks on the seabed. Moreover, research on resilience has led the development of the Resilience Scale for Adult (RSA) assessing resilience factors among adults. The scale was developed based on empirical evidence that existed at the time, it is assumed to be applicable regardless of culture (46, 47). According to the RSA, resilience consists of six factors; perception of self, planned future, social competence, structural style, family cohesion, and social resources (48). People with a higher level of resilience (as per RSA) seem to tolerate stressful life events better (49), whilst individuals reporting fewer resilience resources were more prone to develop psychiatric symptoms when exposed to stressful life events. Thus, resilience resources act as protective factors. With regards to the present study, the results reflect several of the resilience factors of the RSA. As assessed in the factor of Perception of Self (assessing a fundamental confidence in own abilities in solving, managing, and coping in dire events/situations), saturation diving itself can be considered a dire event. In line with the factor of Planned Future (assessing a preference of making plans or setting clear future goals, and having a positive outlook of own future) the divers expressed plans such as early retirement or studying in the chamber to have a way out after their diving career. As to the factor Structured Style (assessing the preference for establishing routines, planning ahead, and approaching tasks in an organized manner), the divers displayed a preference to create personal routines. Further, the factor Social Competence (assessing the ability to create friendships and engage socially, feeling at ease, and being flexible in social interactions or social settings), the divers emphasize this when describing successful divers to be socially competent, showing consideration to one another, and being sociable. Based on the corresponding findings between the descriptions given by saturation divers, and the empirical evidence gained by the work on RSA, it could be recommended to go further with investigating how these resources may prospectively influence the health and career of saturation divers.

The results also indicate that the divers must be flexible to abide by the challenging demands and contrasts in their working and personal lives, e.g., adapting to life at home after being locked away in a rough male-dominated environment, or adapting to the other teammates in the chamber. The results can therefore be seen in accordance with the concept called psychological flexibility (50). Psychological flexibility refers to a human ability to adapt to and recognize shifting situational demands, as well as the ability to shift behavioral repertoire or mindset if required in order to uphold a healthy personal and social functioning. Psychological flexibility may protect a person from depression and anxiety, and be a source to psychological health (50). The divers also highlighted the value of a stable family/home life when adapting to life at home between assignments. Hence, family can be considered an interpersonal resource that supplies the diver with social support, which is considered exceptionally important for maintaining good mental health by enhancing resilience to stress (20, 25, 51, 52).

Autonomy is main factor in reducing work related strain (53), and is considered a central job resource in the JD-R literature (18, 54, 55). However, the tasks performed on the seabed can be described as highly procedural for safety reasons, seeing that small errors may have economic, environmental, and life-threatening consequences. This leaves the divers with little task-related autonomy. Further, the time spent in the chamber system is characterized by little room for privacy, spending several weeks living together in a chamber with the size of a caravan, leaving the divers with limited room for activities of daily living. Interestingly, the divers seem to create some form of autonomy in a seemingly little autonomous supporting work environment by creating unique personal routines in the chamber. Little routines, such as bringing enough of the right sort of tea or hand brewing specific types of coffee, are described as essential when living in the chamber system. The same routines were non-essential to the divers at home. Three of the divers explain that these types of routines can become so important that it may determine whether the day is good or bad. One diver described these routines as a means of establishing meaningfulness in situations that can be perceived as meaningless. This act of creating personal routines can be considered as a form of job crafting, or maybe even “leisure crafting” (56) as it is performed during the off-time in saturation. Job crafting is the act of optimizing of work environment by proactively changing the job demands or job resources, making their job more meaningful or interesting (55). Further, this meaningfulness which the divers create by shaping personal routines, is considered in Work-Related Sense of Coherence (57) to be a motivational component that reflects whether the employee experience own work situation as worthy of involvement and commitment. A work situation experienced as meaningful is shown to enhance the employees engagement (58).

With regards to another interpersonal aspect expressed to be salient, both the team harmony and team cohesion not surprisingly were pointed out to be important determinators for their working lives. A disharmonious or discordant team was considered demanding. As one of the divers explains, there occasionally have been fights in the chamber system. The team climate does not have to be disharmonious to be demanding. One informant explains that small things, such as chewing on the spoon, can become irritating over the weeks in isolation. Similarly experienced by a soviet cosmonaut; “All conditions necessary for murder are met if you shut two men in a cabin measuring 18 feet by 20 and leave them for two months.” (59). A solid group cohesion has countless benefits, e.g., lightening the social monotony of living in an ICE, or creating a safe space for venting frustrations, hence avoiding serious conflicts [(60), p. 56]. A review and analysis of astronauts' and cosmonauts' personal journals (59) show that the participants highest priority before launch was to get along with their crewmates. Their journals show that astronauts and cosmonauts actively behave in a way to maintain interpersonal harmony (by being cooperative, avoiding certain topics in conversation, and other sincere acts of comradeship). Similarly, the informants of the present study highlight that the most important group characteristics in saturation are being considerate and sociable.

Interpersonal trust was considered important by the informants in this study. As one diver points out, when working with a less experienced diver, they must ensure and control that safety precautions are followed so no lives are at risk. Thus, lack of trust can be demanding. Trust, defined as a person's willingness to rely on another individual in a risky situation (61), is developed by ones perception of the other person's qualities (62). Inexperienced divers or divers who show little interest in learning the procedures of the job are experienced as less trustworthy, which increases the demands on more experienced divers who must control the situation. Research has demonstrated that a combination of high job demands and low perceived job control is related to significant strain and negative health effects (53). Similarly, the constant responsibility for the lives of others was considered to be one of many daily hassles among Swedish military veterans, whilst the capability of the unit, and trusting each other were seen as daily uplifts to their work environment (36). A study investigating the effects of trust in high reliability organizations found that higher levels of trust were related to a lower risk perception (63). These findings may explain why danger is not explicitly considered to be one of the main challenges in the working lives of the divers.

### Organizational Demands and Resources

Job insecurity is considered a psychosocial hazard at work (64), and has been increasingly studied in relation to ongoing transformation in organizations and labor markets, leading to increased job instability and flexibility, e.g., in downsizing, when experiencing strong competition in the labor markets, and the use contingent contracts (65). Studies have found job insecurity to negatively impact workers' mental health (66, 67). Further, meta-analysis relates job insecurity to the absence of trust in management, lower organizational commitment, higher turnover intention, and poorer performance (66). A study applying the JD-R model found job insecurity to have a direct and an indirect effect (via burnout) on organizational outcomes such as turnover intentions and commitment (68). As demonstrated above, job insecurity affects not only the individual employee exposed to it, the organization is also negatively affected. However, neither social support from colleagues, management, nor the unions seem to have a buffer effect on this type of stress. Job insecurity is therefore regarded as a stressor to be dealt with independently, and cannot be made less harmful by providing social support (69, 70). As one diver explains, there is little to be done to this particular challenge.

Further, the results show how the divers balance the organizational job resources, namely high wages and leisure, against the high personal demands they encounter in their occupation, such as missing out on big life events, not being able to plan a family holiday, and having a monotonous working day most of them don't experience to be satisfying anymore. In accordance with the JD-R model rewards such as pay might function as job resource, and therefore as a predictor of work engagement (20). In studies that apply the JD-R framework, non-financial rewards such as social support, positive feedback, and developmental opportunities are convincingly documented as job resources that can mitigate demanding work conditions and predict work engagement (18). As explained in Kulikowski and Sedlak (71) the role of monetary rewards in predicting work engagement is undecided. Studies investigating the connection between monetary rewards and work engagement are few, and have reported mixed conclusions. On one hand one study found that positive evaluation of current pay functions as a positive predictor on work engagement (68), and another that the prospect of being rewarded for high job performance correlated positively with work engagement (72). On the other hand, Hulkko-Nyman et al. (73) suggested that salaries was unrelated to work engagement, whilst Kulikowski and Sedlak (71) found no relationship between the commonly used Human Resource Management rewards (salary, benefits, and bonuses) controlled for different job demands and resources. In this study, the high salaries seem to serve as a buffer for the high job demands (22), rather than a direct predictor of work engagement.

Leisure was also pointed out as an important factor for informants. One diver considered leisure to be the best part of the career. Periods of leisure might function as a buffer for the high job demands after 4 or 5 weeks of working. When employees encounter high job demands repeatedly, it's likely that they experience higher levels of strain and should therefore launch into recovery activities. Recovery can be defined as a process of restoring cognitive and energetic resources expended in the work situation (74) where the employee is considered to be recovered when these resources return to baseline (75). Daily recovery during off-time was positively associated with work engagement the next day (74), and better work performance (76). Moreover, leisure may function as a buffer for negative work-family spillover, which has been associated with lower subjective wellbeing (77) and reduced mental health (78–80). It has been suggested that engaging in leisure activities might enhance the subjective wellbeing (81) either by facilitating the work and the family domain, or via leisure satisfaction (82). However, the contrasts in levels of autonomy are great when comparing the divers' lives in saturation and their off-time at home. The divers are fully autonomous when they are not at work, until they are summoned for a new assignment. This way, the leisure during off-time is indirectly controlled or on the leash of the diving companies.

Along with leisure and high wages, the results also show that employment as a commercial saturation diver is experienced as winning the lottery because there are very few jobs and the pay is high compared to inshore diving. This feeling of passing the “eye of a needle” by getting a job in saturation diving might function as a buffer resource in line with the findings of Rennesund and Saksvik (83) who studied collective or organizational efficacy on stressor-strain relationships. Their study found that the feeling of being member of a highly competent organization seems to mitigate the inimical effects of work stressors on the individual employee by providing them with a sense of being “best in class” and a sense of security.

The work environment in saturation diving might be perceived as thwarting rather than supporting need satisfaction. Working as a saturation diver therefore seems to encompass a willingness to periodically renounce personal needs in exchange for major external rewards. Still, throughout the results it is apparent that the divers themselves act in a way to fulfill their needs for relatedness, competence and autonomy (84), as a way to foster “optimal” functioning in a challenging work environment. With regards to relatedness, the divers spend weeks away from family and friends and miss out on big life events such as funerals and their children's birthdays, etc. This way the divers periodically disconnect from the outside world and the people who usually contribute to the fulfillment of the personal need of relatedness. In saturation, the comradery and the social climate within the team are highlighted as essential for a good work situation, comprising both adaptability and consideration from every team member. Being interested in learning is a personal resource that was highlighted by the informants as important for success in their profession. The studying some divers do during their off-time in the chamber may also be considered an act of fulfillment of their need for competence. Ultimately, the need for autonomy is shown by the leisure crafting during their off-time in saturation through which they control their existence by shaping personal routines. This way the divers may avoid need frustration which has been associated with job strain and higher levels of work-related stress (85).

## Strengths and Limitations

To the our best of knowledge, this is the first study investigating factors that are unique to the psychosocial work environment among commercial saturation divers, and can be considered a contribution to fill in the knowledge gap of psychological effects within saturation diving (2). The informants included in the study had extensive experience within this line of work and provided rich descriptions of the working life of saturation divers. By using an already established theoretical framework, the Job-Demands Resources Model, the results add unique information to this specific occupation. However, this study has some limitations. When applying the template analysis to the data, the focus was to capture the specific factors expressed by the divers as either protecting or demanding. This focus may have forsaken other important aspects of the divers' experiences of their work environment. Moreover, the JD-R model is heuristic; it characterizes the job and personal traits that lead to the outcome, but does not explain their origins. Further, studies with an exploratory nature, like the present, are prone to debatable generalizations. Furthermore, the results were based on data from divers with several years of experience within this line of work, which may give rise to bias. The findings may primarily be relevant to people who are comfortable in ICE conditions, due to the possible selection bias of the “survival effect” or the “healthy worker effect” (86). This may also explain how some of the challenges the divers encounter are seen by them as merely daily hassles. The viral pandemic that was raging at the time of the study should also be considered as it -along with low oil prices—puts an extra pressure on the industry. Even though saturation diving for the oil and gas industry may be at risk, the results of this study may be relatable to other occupations characterized as isolated. The obvious links to occupational fields such as space flight and army missions have been discussed earlier. But also, during the pandemic, many people have been forced to work from home, experiencing the demands of decreased freedom. To that end, this current study may provide a bit of insight from the isolated environment that saturation divers embark, when they are called on duty. While this study focused on the identification of demands and resources as described by commercial saturation divers themselves, further longitudinal quantitative research is needed to investigate the potential importance and impact of these factors. To that end, our results provide insight into areas for further investigation.

## Conclusion

The aim of this study was to contribute to fill the identified knowledge gap of the psychological effects of saturation diving (2) by exploring their psychosocial work environment. In answer to the research question: “*Which unique demands and resources are viewed as characteristic among saturation divers working within an isolated and confined environment?”* The informants emphasized an *unpredictable future* as a major demand, due to changes in the oil and gas industry. Another important demand was the *conflict between family and work/leisure* when committing to work for 21–28 days in an isolated environment. The *monotony* of this environment provides an additional challenge. *High wages, long periods of leisure*, and *a prestigious job* provide external resources in order to maintain this work, while personal resources such as an *interest in learning, mental endurance* and *flexibility*, and *keeping up small personal routines*, play important roles. Also emphasized are the quality of team climate—described with features such as being *sociable and considerate*, having *a dark sense of humor*, and having *trust* in one another. The results from this study indicate a need for further research into resilience resources and their application in ICE occupations like commercial saturation diving.

## Data Availability Statement

The datasets presented in this article are not readily available because data are based on qualitative interviews with informants from a small group of workers. We can provide additional quotes that correspond to the themes described in results. Requests to access the datasets should be directed to jonas.vaag@nord.no.

## Ethics Statement

This project was registered and approved by the Norwegian Centre for Research Data (NSD). The patients/participants provided their written informed consent to participate in this study.

## Author Contributions

SR and JV planned the project with assistance from IE. SR and JV developed the interview guide. SR conducted the interviews. Analysis was done by SR with assistance from JV. SR drafted the outlines of the manuscript, of which all authors contributed to the final draft. All authors contributed to the article and approved the submitted version.

## Funding

IEs contribution to the article was funded by the Norwegian Research Council and Equinor on behalf of PRSI Pool through the Large-scale Programme for Petroleum Research (PETROMAKS2), project no. 28042.

## Conflict of Interest

The authors declare that the research was conducted in the absence of any commercial or financial relationships that could be construed as a potential conflict of interest.

## Publisher's Note

All claims expressed in this article are solely those of the authors and do not necessarily represent those of their affiliated organizations, or those of the publisher, the editors and the reviewers. Any product that may be evaluated in this article, or claim that may be made by its manufacturer, is not guaranteed or endorsed by the publisher.
